# Flexible Multilayer Plasmonic Films for Biosensing and Photoemitting Applications

**DOI:** 10.1021/acsomega.4c07333

**Published:** 2025-02-11

**Authors:** Le Thi Quynh, Chang-Wei Cheng, Shangjr Gwo

**Affiliations:** † Department of Physics, National Tsing-Hua University, Hsinchu 30013, Taiwan; ‡ Department of Photonics, National Yang Ming Chiao Tung University, Hsinchu 30010, Taiwan; § Institute of Nanoengineering and Microsystems, National Tsing-Hua University, Hsinchu 30013, Taiwan

## Abstract

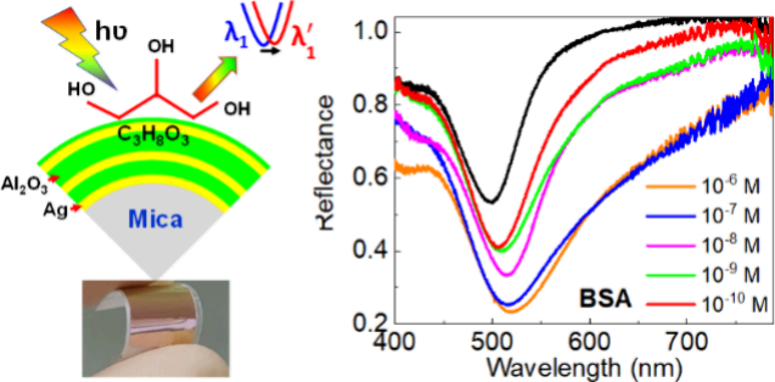

Flexible plasmon metasensor devices describe the use of multiple Ag/Al_2_O_3_/mica layers for tunable plasmonic resonances and are a promising research direction. Here, we report on a flexible Ag/Al_2_O_3_/mica multilayer platform and its excellent performance on flexible biosensors and photon-emitting devices. In our approach, muscovite (mica) was adopted as a single-crystal substrate due to its optical transparency and mechanical flexibility. The Ag/Al_2_O_3_/mica multilayer film is characterized by X-ray diffraction and transmission electron microscopy. Optical, plasmonic, and biosensing studies of Ag/Al_2_O_3_/mica multilayers are performed for detailed understanding. A combination of optical absorption, numerical simulations, and optical reflectance measurements has confirmed the biosensor performance. Two kinds of flexible plasmonic device applications are reported here, including (1) plasmonic biosensors with high refractive index sensitivities and (2) significantly enhanced spontaneous photoluminescence (PL) of monolayer tungsten disulfide (WS_2_) spectra. We found that the PL emission under 0.4 mm^–1^ curvature bending state increased to 16% compared to the unbent state and redshift of 60 meV/% strain in the emission of WS_2_ monolayer. Furthermore, the Ag/Al_2_O_3_/mica multilayer film displays robust stability and strong endurance up to a bending curvature of 0.4 mm^–1^. This study shows great potential to be used for biosensors and flexible optoelectronics.

## Introduction

Nanomaterials and nanostructures have caught great attention in previous decades due to their fascinating optical and physical properties and potential applications.^1−13^ The coupling of light and nanostructures results in a broad spectrum of intriguing functionalities, such as propagating surface plasmon polaritons (SPPs) at the metal–dielectric interface and localized surface plasmons (LSPs) in the metal nanostructure, and offers tremendous opportunities for next-generation optoelectronic devices. Furthermore, the surface plasmon dispersion and resonance can be effectively changed and tuned by the surrounding dielectric environment or the size and shape of the metal nanostructure.

Metal insulator heterostructures and multilayer films have drawn great scientific interest since those materials provide promising couplings between electric and optical order parameters and the potential to manipulate one through the other. Thus, such multilayer systems can exhibit unique properties including epsilon-near-zero (ENZ) and flexible optomechanical manipulation creating a huge playground to discover new emergent phenomena.^14−22^ Besides, two-dimensional (2D) semiconductors have caught great attention in previous decades due to their fascinating physical properties and potential optoelectronic applications. The strong coupling between plasmon resonance and excitons can result in a significant enhancement of the photoluminescence of 2D monolayers. Prominent examples are negative refraction,^23^ lasing,^24^ enhanced spontaneous emission,^25−27^ supper resolution,^28,29^ light-harvesting system,^30^ and biosensors.^31^

The flexible surface plasmon biosensors have drawn great scientific interest since those materials provide promising couplings, stability, functional metamaterials, and the potential to manipulate one through the other.^32−35^ To obtain such intriguing phenomena, precise control of the single-crystal substrate surface condition on the atomic scale is extremely crucial and is the first focal key to control the noble metal multilayer growth at the atomic scale. The mica substrate is chosen due to its large available size, atomic flatness, high thermal stability, optical transparency, and material flexibility.^36−39^ Furthermore, the weak van der Waals (vdW) interaction at the interface between the metal film and the mica substrate can minimize the substrate clamping effects, which might induce some detrimental effects, compromising the functionalities of the thin films. In the case of growing ultrathin metal layers for tunable plasmonic applications, we also find that the approach of vdW heteroepitaxy on mica can also prevent the common dewetting issue of metal films without the need for an additional wetting layer between the metal film and the substrate.^36,39^ In the past decade, plasmonic metasensors had strong light confinement and tunable plasmonic resonance and have become an exciting new paradigm with the potential to impact the economical fabrication of biosensor devices.^40,41^

In this study, the Ag/Al_2_O_3_ multilayers serve as nanocavities, with alternating layers Ag and Al_2_O_3_, that exhibit a remarkable biosensing performance based on ENZ cavity resonances and high-quality factors of their surface state. Thanks to the advances of modern growth techniques, such as thermal evaporation and pulsed laser deposition, we are now able to control the growth at the atomic scale; we fabricate the Ag/Al_2_O_3_ multilayer nanomaterials, in which the thin Ag film and Al_2_O_3_ dielectric film is alternatively, for plasmonic induced biosensing with high efficiency. The crystallinity and microstructures of the Ag/Al_2_O_3_ multilayer film on mica are shown by a combination of high-resolution X-ray diffraction (HRXRD) and transmission electron microscopy (TEM) measurements. The properties of the Ag/Al_2_O_3_ thin film were characterized to ensure its functionality. In such multilayer nanomaterial systems, the sharp interfacial Ag film, the outcome of plasmon resonance at the visible range, can lead to enhanced sensing performances and photoluminescence of WS_2_ monolayers. Our results represent an important step toward establishing flexible biosensors based on Ag/Al_2_O_3_/mica multilayer thin films. Such an approach not only sheds light on the potential of room temperature optoelectronic emitting but also triggers novel inspiration for stability and flexible plasmonic biosensing devices. The demonstrated enhanced sensor performance makes the system promising for practical integrated biosensing platforms and plasmonic optic on a chip.

## Experimental Section

### Sample Growth

Ag/Al_2_O_3_/mica multilayers were performed on single-crystal mica substrates using a thermal deposition system with a base pressure ∼1 × 10^–8^ Torr. These films were grown by using high-purity Ag metal sources (99.99%) under 150 °C and then cooled down to room temperature in 30 min. Then, the Al_2_O_3_ layer was immediately deposited by using an atomic layer deposition (ALD) system at 150 °C. The thicknesses of these films were obtained by a thickness sensor inside the growth chamber.

#### Preparation of Aqueous Solution for Biosensing on Ag/Al_2_O_3_/Mica Multilayers Surface

In order to investigate the sensing performance of Ag/Al_2_O_3_/mica multilayer films, hemoglobin and bovine serum albumin (BSA) were purchased from Merck Ltd.

The glycerol solutions with different weight percentage concentrations were prepared in distilled water. Different concentration of BSA solution (10^–6^, 10^–7^, 10^–8^, 10^–9^, and 10^–10^ M) was freshly prepared with ultrapure Milli-Q water, while the hemoglobin with varying concentration of 10^–5^, 10^–6^, 10^–7^, and 10^–8^ M was prepared with aqueous solution. Then, the films were soaked in biosolution for 3 min before measuring reflectance spectra.

### Structural Characterization

The surface morphology of thin films was examined by atomic force microscopy (AFM) using the tapping mode. High-resolution X-ray diffraction (XRD, Bede D1 diffractometer) was employed to examine the crystal quality. The specimens for transmission electron microscopy (TEM) in the cross-sectional configuration were prepared by using a focused ion beam (FIB, FEI Helios Nanolab 600i). The Ag/Al_2_O_3_/mica multilayer films were characterized by using a high-resolution transmission electron microscope (JEOL JEM-F200).

### Optical Measurements

We used a halogen white light (Ocean Optics HL-2000-CAL) as the incident light to excite the Ag/Al_2_O_3_/mica multilayers film. The reflection light was collected by the ×50 objective lens (Olympus, N.A. = 0.42) and aligned by a lens onto an imaging CCD or the entrance slit of UV–vis spectrometer (Andor SR-500i-A, equipped with a DU401A-BV CCD) for imaging or spectral analysis. The photoluminescence (PL) emission measurement was carried out at room temperature. The multilayer film was excited by a 532 nm diode-pumped solid-state laser through a ×50 objective lens (N.A. = 0.42, HLB-M Plan Apo Series, Shibuya Optical Co.). The PL signal was acquired using a micro-Raman microscope (RAMaker, ProTrusTech). The Raman signal was collected by a fiber-coupled spectrometer (MS2004i, SOL Instruments).

### Ellipsometry Measurements

The dielectric function (ε_1_, ε_2_) of thin films was measured by using a spectroscopic ellipsometer (m2000, J. Woollam Co.). The Ag/mica and Ag/Al_2_O_3_/mica multilayer films were measured under three different incident and collection angles: 65, 70, and 75° with respect to the normal direction of the film surface. The dielectric function of thin films was obtained by fitting with the Drude–Lorentz and B-spide models. For investigating the absorption band of Ag multilayer films, we adopted the Hitachi, U-4100 UV–visible NIR spectroscopy.

### FDTD Simulations

A commercial software package (FDTD Solutions, Lumerical Inc.) was used to simulate the optical properties of the silver multilayer films. In these simulations, the permittivity of the metal film was adopted from our experimental results.

## Results and Discussion

**Figure 1:**
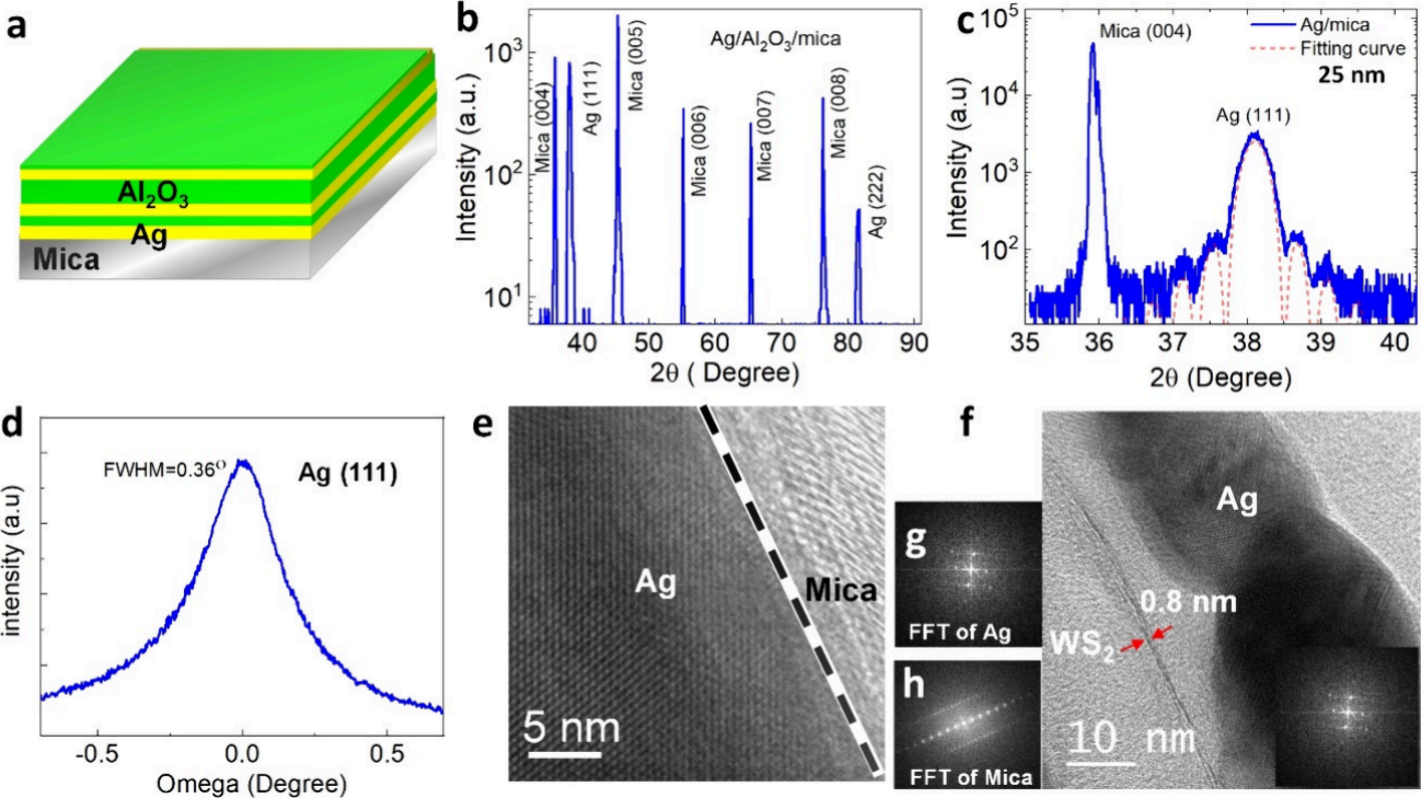
Structural characterization of Ag/Al_2_O_3_/mica multilayer films on mica. (a) Schematic of silver multilayers Ag/Al_2_O_3_ structures including 3 Ag layers and 3 Al_2_O_3_ layers. (b) X-ray diffraction 2θ–θ scan showed only Ag (111) and Ag (222) peaks on mica. (c) High-resolution XRD scan at Ag (111) peak. (d) Rocking curve at the Ag (111) diffraction peak. (e) Cross-sectional TEM image showing the crystalline structure and sharp interface of Ag (111) film on mica. (f) High-resolution TEM at the interface showing WS_2_ on multilayer film. (g) and (h) Corresponding FFT patterns of Ag and mica.

In this study, Ag/Al_2_O_3_/mica multilayer thin films of Ag and Al_2_O_3_ were grown alternatively on (001) muscovite mica (mica) by thermal evaporation under high-vacuum conditions and atomic layer deposition. Figure 1a shows the schematic of the Ag multilayer structures. The structural details were determined by high-resolution X-ray diffraction. Figure 1b shows a typical out-of-plane 2θ–θ scan of a Ag/Al_2_O_3_/mica multilayer film. Only Ag (111) and Ag (222) diffraction peaks can be detected, illustrating the good crystalline quality of thin film without other secondary phases. The lattice constant of Ag calculated from the XRD angles is 2.36 Å, which is very close to the bulk value (amcsd-0011135). Therefore, the film is almost fully relaxed as expected for van der Waals epitaxy. Furthermore, the HRXRD curve around mica (004) shown in Figure 1c displays clear thickness fringes, indicating a sharp interface and smooth surface of the Ag film. Rocking curve measurements were used to obtain more information about the crystalline quality. The full-width at half-maximum (fwhm) of the Ag (111) diffraction peak is 0.36°, suggesting a superior crystalline quality of the Ag film (as shown in Figure 1d). To investigate the microstructure and further confirm the monolayer of WS_2_ on a Ag/Al_2_O_3_/mica multilayer film, high-resolution transmission electron microscopy (TEM) was carried out. In Figure 1e,f, the high-magnification cross-sectional TEM image shows a flat interface of Ag on mica and WS_2_ monolayer plated on the Ag/Al_2_O_3_/mica multilayer structure surface. The thickness of the 0.8 nm WS_2_ layer was identified on the interface and corresponding to the monolayer of WS_2_. The corresponding selected area electron diffraction patterns were captured near the interface between the Ag thin film and muscovite substrate, as shown in Figure 1g,h. The out-of-plane relationship can be obtained as (111)_Ag_ || (001)_mica,_ in good agreement with the XRD result. In addition, the sharp interface of Ag and Al_2_O_3_ was confirmed by AFM and SEM, as shown in Figures S1 and S2. The Ag and Al_2_O_3_ layers are visible as bright and dark bands, respectively.

**Figure 2:**
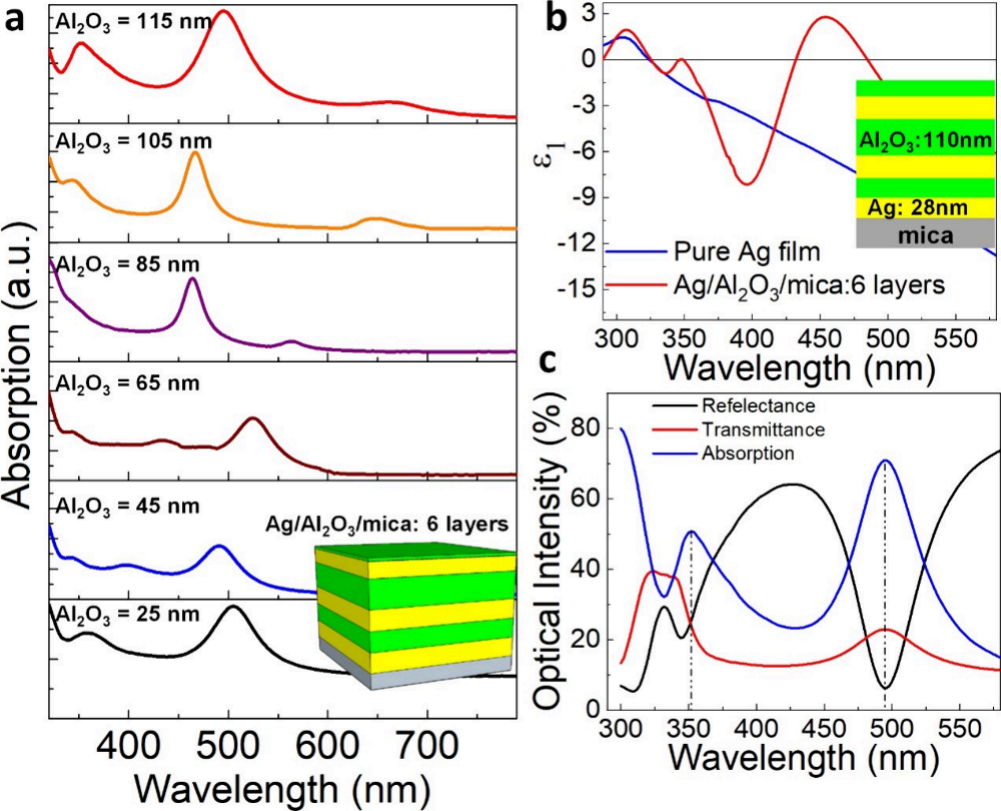
Optical characterization of Ag/Al_2_O_3_/mica multilayer films on mica. (a) Tunable plasmon resonance of Ag/Al_2_O_3_/mica multilayer via controlling the thickness of the Al_2_O_3_ layer. (b) Comparable the dielectric function (ε_1_) of pure Ag and Ag/Al_2_O_3_/mica structures. (c) Measured optical properties of Ag/Al_2_O_3_/mica multilayers.

After confirming the microstructures, now we focus on the benefits of Ag/Al_2_O_3_/mica multilayer flexible films being tunable plasmonic resonance and epsilon-near-zero behavior in the visible regime. Thanks to its excellent optical properties in the visible regime, Ag is a promising plasmonic candidate for plasmonic hybrid systems. On the other hand, mica opens a new era for epitaxial thin film applications that can be bendable. The optical properties of the films were investigated by the ellipsometry spectrometer and U-4100 UV–visible NIR spectroscopy, Hitachi. The metal–dielectric–metal interlayer system exhibits epsilon-near-zero at the visible range, and cavity resonance can be tunable when increasing the thickness of the Al_2_O_3_ dielectric layer. For instance, we can control the thickness of the Ag and Al_2_O_3_ dielectric layers to tunable plasmonic resonance. Controlling the thickness of films in the multilayer systems is also important to determine whether either metallic (ε < 0), dielectric (ε > 0), or near-zero (ε ∼ 0) if losses are sufficiently small. Such ENZ resonances have been demonstrated as the resonance tunneling of photons in double-barrier quantum well systems. ENZ resonance can be found at 327 nm as Ferrell–Berreman mode in Ag. The mechanism of electron tunneling phenomena and ENZ occurring in multilayer films has been demonstrated in previous studies.^18,20,42^ Furthermore, the sample is flexible, as shown in Figure S1a, where the sample is bent up to 0.4 mm^–1^ bending curvature. All of these advantages suggest that the growth of functional noble metal multilayers on mica is a new direction to engineer flexible plasmonic and flexible biosensing for practical applications. Figure 2a displays the absorption of Ag/Al_2_O_3_/mica multilayer thin films with varying thickness of the Al_2_O_3_ layer. The absorption of multilayers has displayed the Ferrell–Berreman Ag mode at 325 nm and nanocavity resonances at 350 and 500 nm, respectively. Figure 2b shows the real part of the effective permittivity curve corresponding to the Ag (25 nm)/Al_2_O_3_ (105 nm)/Ag (25 nm)/Al_2_O_3_ (40 nm)/Ag (25 nm)/mica film. The near-zero effective permittivity position was found to occur at λ = 370 and 500 nm wavelength. Furthermore, we obtained two absorption peaks in Ag/Al_2_O_3_/mica multilayer film and are close to near-zero points of effective permittivity position. The absorption intensity has significantly enhanced to 70% at 498 nm wavelength, as shown in Figure 2c, for Ag/Al_2_O_3_/mica multilayers. In addition, Ag/Al_2_O_3_/mica multilayer films remain very stable in ambient environments due to the protection layer of surface oxide (Al_2_O_3_), as shown in Figure S2, suggesting the long lifetime plasmon of Ag multilayer films. To demonstrate the angular dependence of Ag/Al_2_O_3_/mica multilayer thin films, we investigated the home-built angle-resolved reflectance measurement.^9^ In Figure S3, we show the measured SPP resonance phase shift spectra on bent surfaces using angle-resolved reflectance with different bending curvatures, including 0.1, 0.13, 0.2, 0.27, and 0.4 mm^–1^. We note that the bending curvature is represented by 1/*R* in which *R* is the bending radius. The SPP resonances get the red shift when the incident angle changes from the normal plane (0°) to 40°. The results showed only small changes in intensity of resonance peaks and the resonance phase remains during the bending state up to 0.4 mm^–1^.

**Figure 3:**
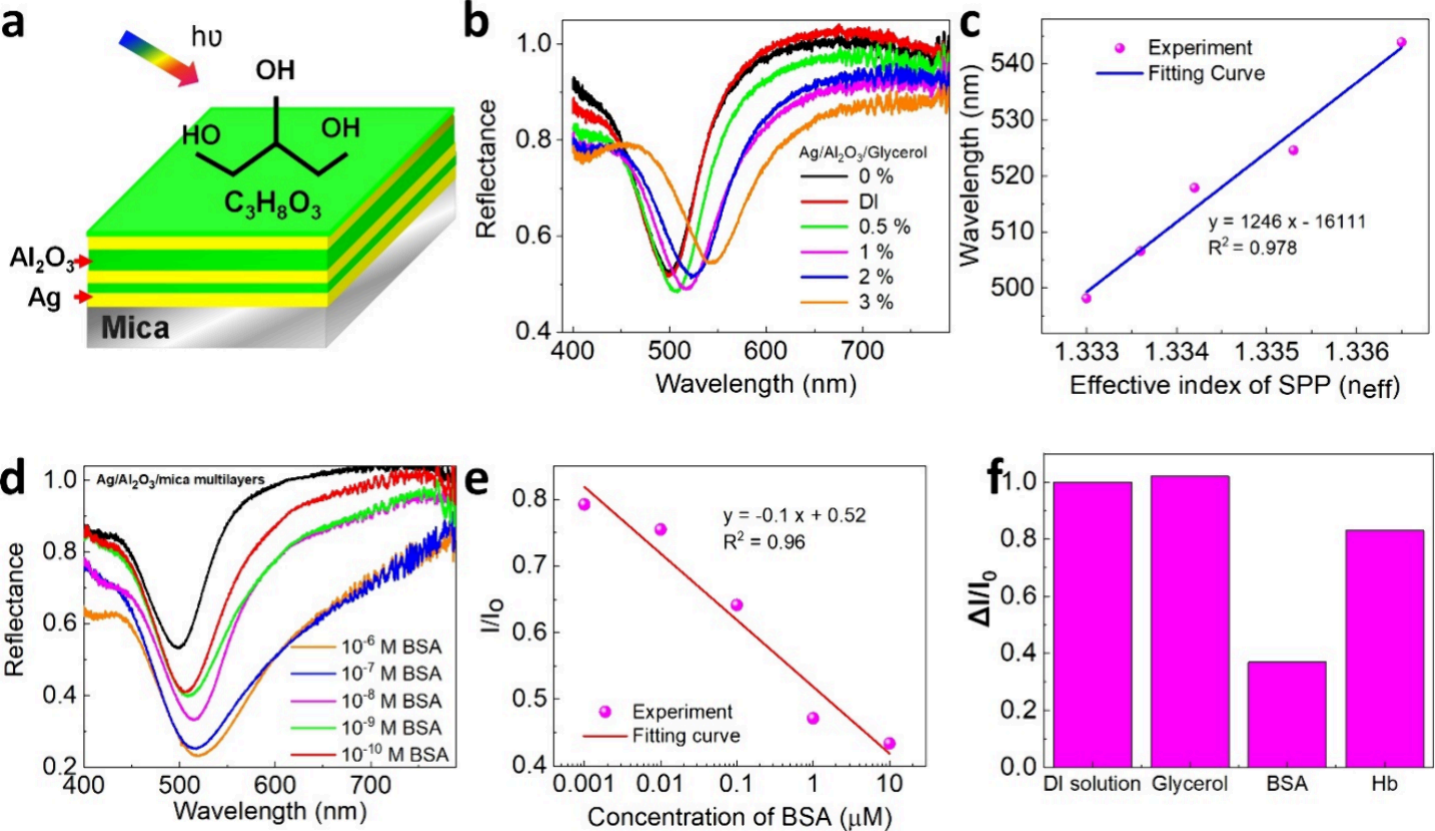
Performance of biosensor on Ag/Al_2_O_3_/mica multilayer films on mica. (a) Schematic of flexible sensing structure. (b) Measured reflectance spectra as a function of glycerol. (c) Wavelength shift for the different effective index of glycerol. (d) Measured reflectance spectra as a function of BSA aqueous solutions. (e) Change in BSA intensity (*I*/*I*_o_) for different concentrations of BSA analyte solutions. (f) Change of intensity for different protein solutions.

Developing multilayer surface plasmon films has emerged as a nanometric light confinement and waveguide based on metal–dielectric–metal interlayers. Having demonstrated the structural and optical properties of the Ag/Al_2_O_3_/mica multilayer films, we next turn to examine the refractive index sensor based on glycerol, bovine serum albumin (BSA), and hemoglobin (Hb) proteins. BSA, glycerol, and hemoglobin emerged as potential bioproteins in the sensing fields. Figure 3a displays the schematic of biosensing. For this purpose, we have measured the reflectance of SPP resonance when injecting different amounts of glycerol, BSA, and Hb on top of the multilayer film. Aqueous solutions of glycerol with different ratios of 0.5, 1, 2, and 3% were manually infected on the surface of Ag/Al_2_O_3_/mica multilayer films; then, the corresponding refractive index change was obtained as a shift in the reflectance spectrum. Figure 3b displays a red-shifted resonance wavelength when increasing from 0.5 to 3% of glycerol. The resonance has a significant shift up to 37 nm corresponding to the ratios of 3% glycerol, and the refractive index sensitivities can be estimated for each spectral position, and the highest value is 16,833 nm/RIU (RIU denotes the refractive index unit). In addition, the highest figure of merit (FOM) for plasmonic sensing is 195 at 518 nm (more details are shown in Table S1). Moreover, the sensing performances on Ag multilayer films show better performance than those of pure Ag/mica films (Figure S4). Under illumination, the cavity resonances can be excited by resonant tunneling through the metal interface resulting in the red-shifted reflectance spectra.^18^ A good linear relationship between the experiment and fitting curve with a correlation coefficient (*R*^2^) of 0.978 is obtained (Figure 3c). In addition, to provide a better insight into the sensor’s performance, we also experimented with BSA target (molecular weight, 665 Da), as shown in Figure 3d. We observed not only the wavelength shift but also significant change in the intensity of reflectance resonance with different molecular concentrations of 10^–6^, 10^–7^, 10^–8^, 10^–9^, and 10^–10^ M. The SPP resonance displays a red shift owing to the surface chemistry. To quantify such variations, a linear function is fitted on the data points, as shown in Figure 3e. The results also reveal that the wavelength shift varies linearly with concentration, decreasing with the number of molecules on the surface. Besides, to investigate the selectivity performance device, hemoglobin also has been examined, as shown in Figure S5. The comparisons of this method with previously reported works are summarized in Table S2. Moreover, the selectivity is one of the key factors for promising sensing platforms. Figure 3f shows the *I*/*I*_o_ decrease remarkably for the BSA analyte solution.

**Figure 4:**
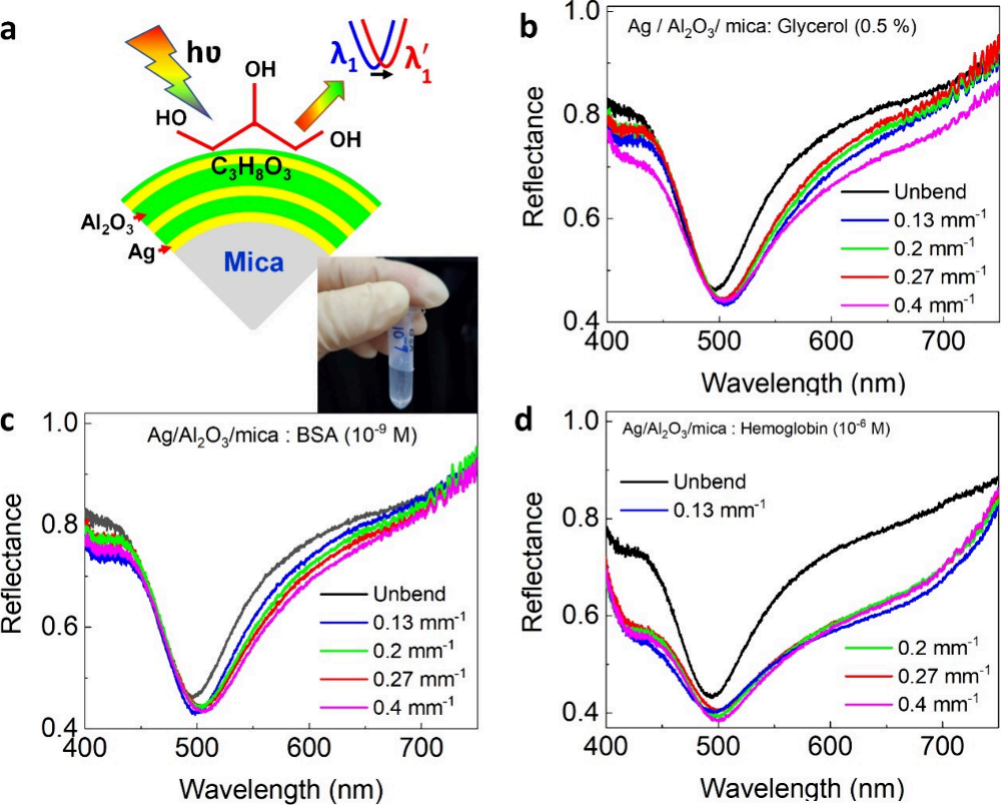
Biosensing performance under bending condition. (a) Schematic of flexible sensing structure. (b) Measured reflectance spectra as a function of glycerol (0.5%) under bending condition. (c) Measured reflectance spectra as a function of BSA (10^–9^ M) under bending condition. (d) Measured reflectance spectra as a function of hemoglobin (10^–6^ M) under bending condition.

To demonstrate the potential of flexible plasmonic sensing using the Ag/Al_2_O_3_/mica multilayers films, the plasmonic sensors under bending conditions were carried out, as shown in Figure 4. Figure 4b–d shows the change of reflectance spectra collected with glycerol, BSA, and hemoglobin as a function of the bending radius under halogen light irradiation. It is clear that the resonance peak is excellently retained even down to 0.4 mm^–1^ bending curvature with a small change in the fwhm peak resonances. This behavior suggests that the microstructure of the multilayer films and thus the sensing properties were not altered in the bending condition, which is attributed to high quality of Ag film and flexible features of mica substrate. Based on the sensing performance, the present work demonstrates that the Ag/Al_2_O_3_/mica multilayer film-based plasmonic devices can be used as flexible plasmonic biosensor platform.

**Figure 5:**
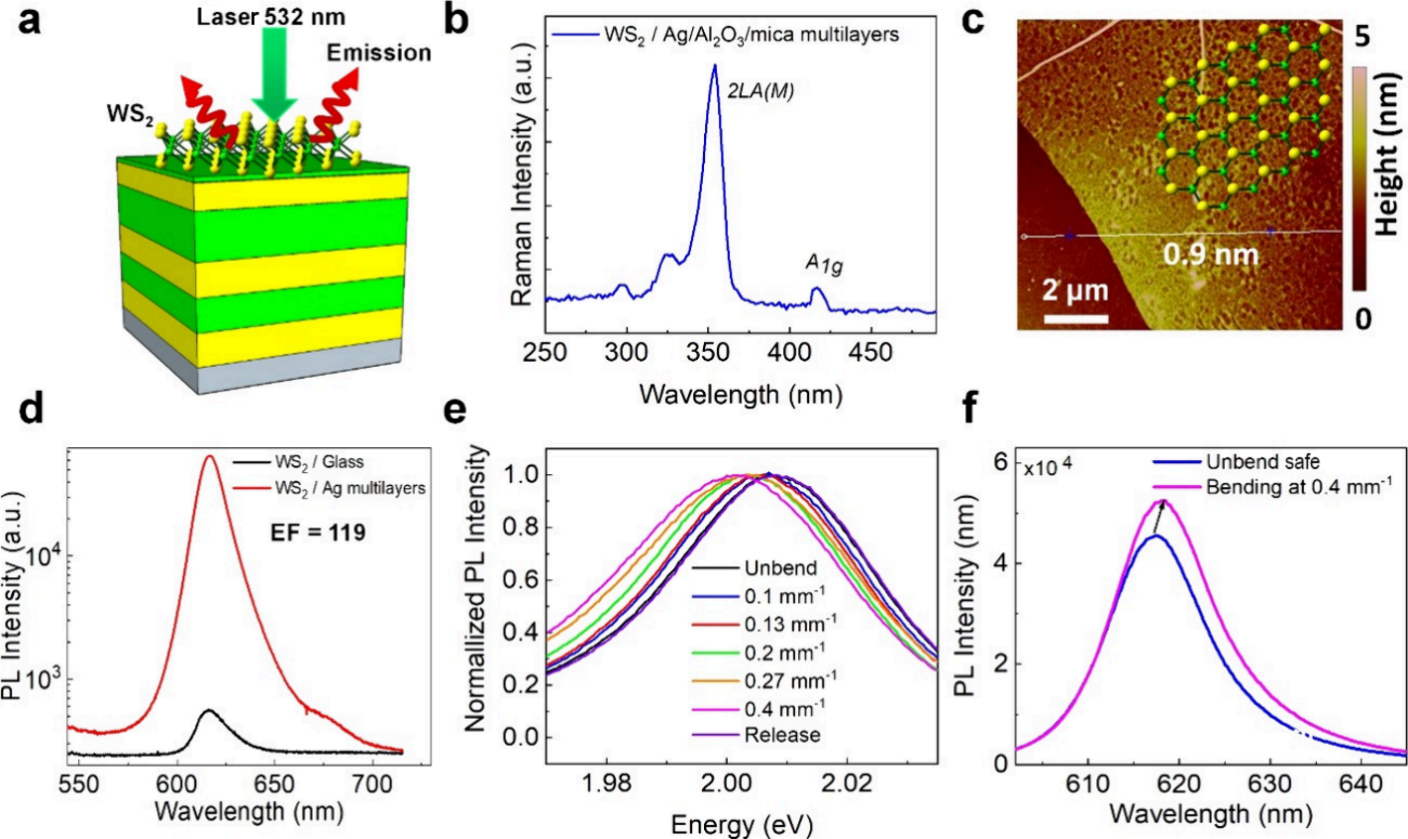
Spectra of photoluminescence (PL) of WS_2_ on Ag/Al_2_O_3_/mica multilayer films. (a) Schematic of WS_2_ on Ag/Al_2_O_3_/mica multilayer film. (b) Raman spectrum of WS_2_. (c) AFM image of WS_2_ monolayer. (d) PL emissions of WS_2_/Ag/Al_2_O_3_/mica multilayers and WS_2_/glass. (e) Normalized PL of WS_2_/Ag/Al_2_O_3_/mica multilayers at different curvature bending states. (f) PL emission spectra of WS_2_/Ag/Al_2_O_3_/mica multilayer film under 0.4 mm^–1^ bending and unbend states.

Plasmon-enhanced absorption and emission have attracted a lot of scientific interest over the past decade. Thus, Ag/Al_2_O_3_/mica nanocavities resonance could confine the light below diffraction limitation, appearing super strong near filed electric field under surface electron resonance.^43^ On the other hand, atomically thin transition metal dichalcogenide (TMDC) on a flexible substrate exhibits unique optical properties, especially a TMDC monolayer, and offers tremendous opportunities for next-generation optoelectronic devices. One of the applications of noble metal multilayers is enhanced photoluminescence of the TMDC monolayer. Figure 5a shows the schematic of WS_2_ on Ag multilayer structures and optical images of transfer samples. For this application, we adopt monolayer tungsten disulfide (WS_2_), an atomically thin transition metal dichalcogenide semiconductor, as the 2D analyte to quantify spontaneous PL performance for Ag/Al_2_O_3_/mica multilayer thin films. A large area of monolayer was transferred on top of the grating structures using a home-built transfer system.^5,9^ As shown in Figure 5b, the AFM image indicates a smooth surface with a height of WS_2_ is ∼0.9 nm, suggesting that a monolayer of WS_2_ can successfully transfer to Ag/Al_2_O_3_/mica multilayers interface agrees well with TEM image results, as shown in Figure 1f. Figure 5c shows the room temperature of the Raman spectrum of the WS_2_ under 532 nm laser excitation. In this spectrum, the longitudinal acoustic mode (*E*^1^_2g_) and out-of-plane vibration mode (*A*_1g_) can be identified. A frequency separation of 63.8 cm^–1^ between *E*^1^_2g_ and *A*_1g_ confirms again the monolayer configuration. The measured PL emission of the WS_2_ on Ag/Al_2_O_3_/mica multilayer thin films was increased up to 119 times, as shown in Figure 5d. Having demonstrated the structural and optical properties of the Ag/Al_2_O_3_/mica multilayer films, we next examined the most unique feature that this system has to offer, the effect of flexibility. Figure 5e shows the results of bending tests carried out on the WS_2_/Ag/Al_2_O_3_/mica multilayer films. The sample has been bent with different bending curvature states ranging from 0.1, 0. 13, 0.2, 0.27, and 0.4 mm^–1^, respectively. We have observed a 60 meV/% redshift of the PL peak with increasing bending curvature. When the sample is released from the bending state, the photoemission properties are restored to nearly the initial state. In addition, we found that the PL signal under 0.4 mm^–1^ curvature bending state increased to ∼16% (1.4 order) compared to the unbend state, as shown in Figure 5f. More detailed comparisons of the strain-dependent PL emission of WS_2_/Ag/Al_2_O_3_/mica multilayer films are presented in Table S3 and Figure S6.

## Conclusions

In conclusion, we have fabricated flexible Ag/Al_2_O_3_/mica multilayer films and demonstrated a remarkable sensitivity of biosensing. The Ag/Al_2_O_3_/mica system with a thickness can be well-controlled and exhibits strong surface plasmon resonance in the visible regime. The strong surface plasmon polariton of Ag/Al_2_O_3_/mica multilayer films has been obtained via transmission measurement and angle-resolved reflectance measurement. The Ag/Al_2_O_3_/mica multilayers show a remarkable sensing performance. The WS_2_/Ag/Al_2_O_3_/mica exhibits a significant enhancement in photoluminescence emission compared to the WS_2_/glass substrate. Using the Ag/Al_2_O_3_/mica multilayer film, we are able to investigate protein detection with high sensitivity, stability, and selectivity of glycerol, BSA, and Hb biosolutions. Our work demonstrates that the Ag/Al_2_O_3_/mica multilayer films can be a potential platform of promising flexible, efficient, high sensitivity, and stability plasmonic biosensing.

## Supporting Information Available

Flexible multilayer plasmonic films for biosensing and photo emitting applications; description of spectroscopic ellipsometry (SE) measurement; optical image bending of Ag/Al_2_O_3_/mica multilayers and its morphology, dielectric function; SEM images of Ag/Al_2_O_3_/mica multilayer film; experiment angle-resolved reflectance measurement; optical images of monolayers WS_2_; and sensing performance and comparison of sensing performance with previous works (PDF)
