# Supplementary material for: Flexible Multilayer Plasmonic Films for Biosensing and Photoemitting Applications
Source: ACS Omega. 2025 Feb 11;10(7):6586–92. doi: 10.1021/acsomega.4c07333 (PMC11865980; doi:10.1021/acsomega.4c07333)
Supplement: Supplementary file 1 — ao4c07333_si_001.pdf [file ao4c07333_si_001.pdf]

Supporting Information for

Flexible Multilayer Plasmonic Films for Biosensing and Photoemitting Applications

Le Thi Quynh,[1] * Chang-Wei Cheng,[1] and Shangjr Gwo[1,2,3]

[1]Department of Physics, National Tsing-Hua University, Hsinchu 30013, Taiwan.

[2]Department of Photonics, National Yang Ming Chiao Tung University, Hsinchu 30010, Taiwan

[3]Institute of Nanoengineering and Microsystems, National Tsing-Hua University, Hsinchu 30013, Taiwan

Corresponding Authors

Le Thi Quynh

Email: lequynhcn@gmail.com

Additional Experimental Details:

Spectroscopic Ellipsometry (SE): The SE measurements were performed using a Woollam M-2000 spectroscopic ellipsometer. The focusing probe attachment provides an incident spot size of ~300 μm. The Ag/Al_2O_3/mica multilayers were measured under three different incident and collection angles, including 60°, 65°, and 75° with respect to the normal plane of the film surface. The collected wavelengths range from 200 nm to 1500 nm. For data modeling and analysis, the Complete EASE software was used to fit the ellipsometry results using the Drude-Lorentz model, which is expressed as

$$\varepsilon(\omega) = \varepsilon_1(\omega) + i\varepsilon_2(\omega) = \varepsilon_{\mathrm{b}} - \frac{\omega_{\mathrm{p}}^2}{\omega(\omega + i\gamma_{\mathrm{p}})} + \sum_{n=1}^{2} \frac{f_n \omega_n^2}{\left(\omega_n^2 - \omega^2 - i\omega\gamma_n\right)}$$

where ε_{b} is the polarization response from the core electrons (background permittivity), ω_{p} is the bulk plasmon frequency, γ_{p} is the relaxation rate (electron-electron scattering loss), f_n and ω_n are the strengths and resonant frequencies of interband transitions, and γ_n are the damping rates of interband transitions. [8]

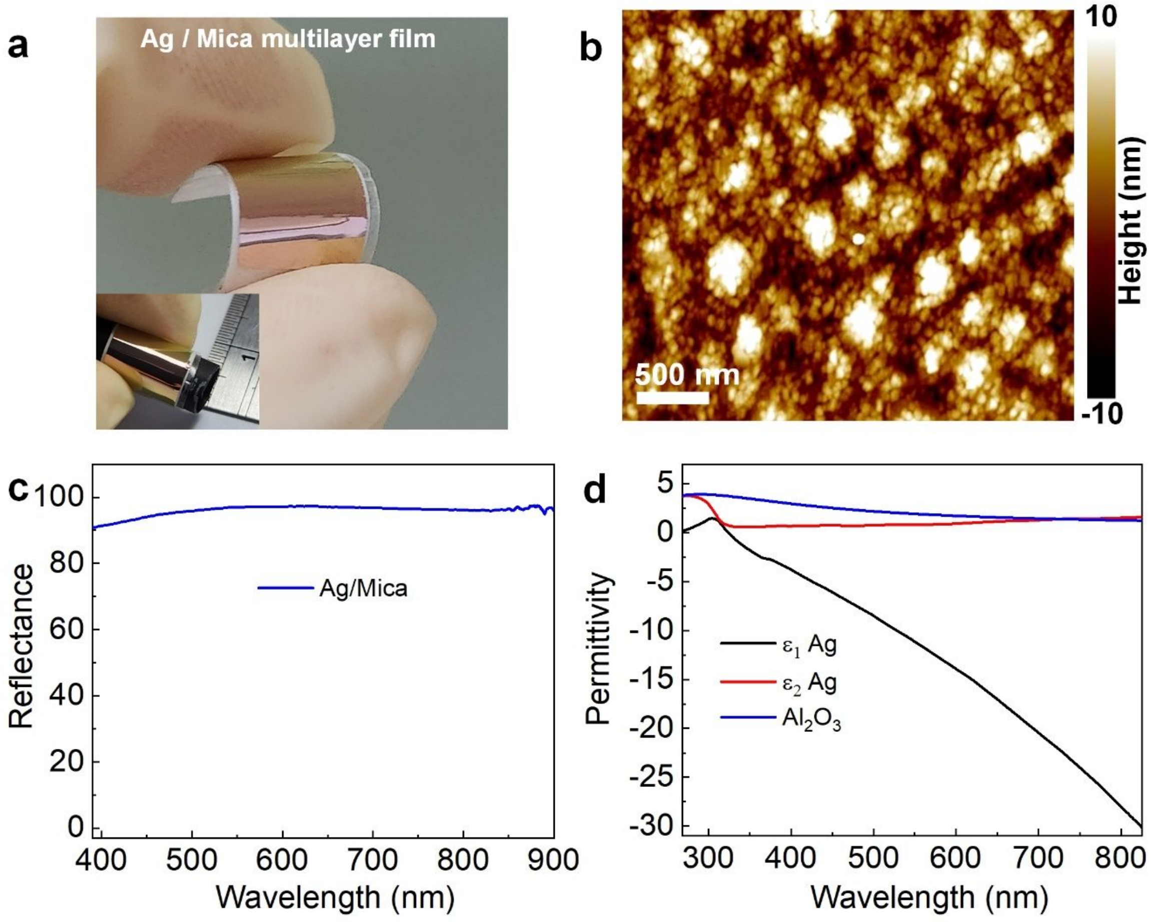

Figure S1. (a) photograph of a flexible Ag / Al_2O_3 multilayers film on mica. (b) Atomic force microscopy (AFM) image (area: 5 × 5 μm^2) of Ag / Al_2O_3 / Mica multilayers. (c) Reflectance spectrum of Ag/ Mica film. (d) the permittivity of Ag and Al_2O_3 films.

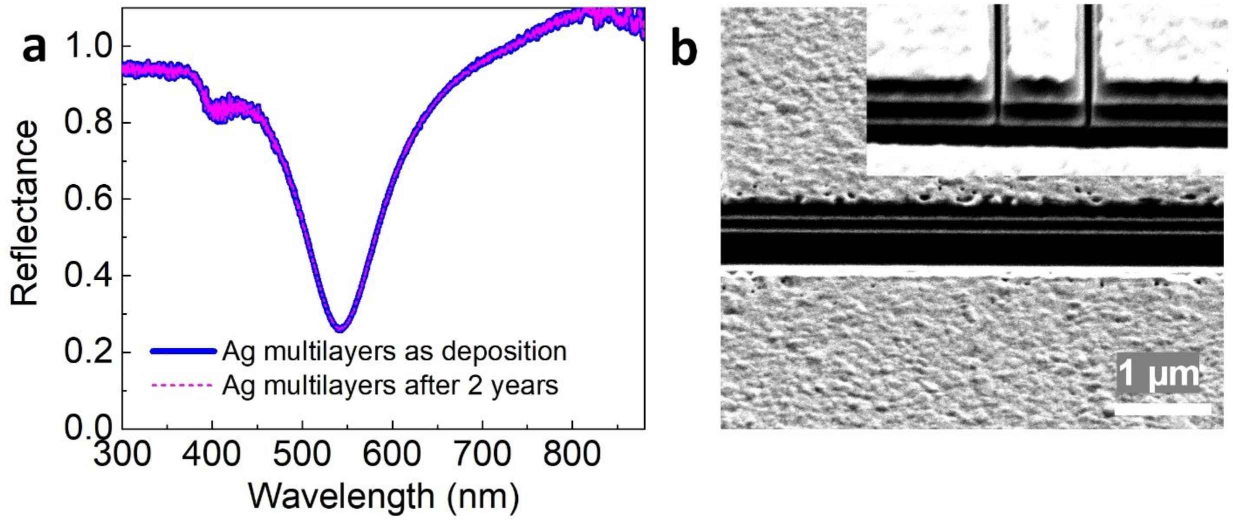

Figure S2. (a) Experiment reflectance of Ag / Al_2O_3 / Mica multilayers films as deposition and after 2 years deposition. (b) Cross - section of scanning electron microscope (SEM).

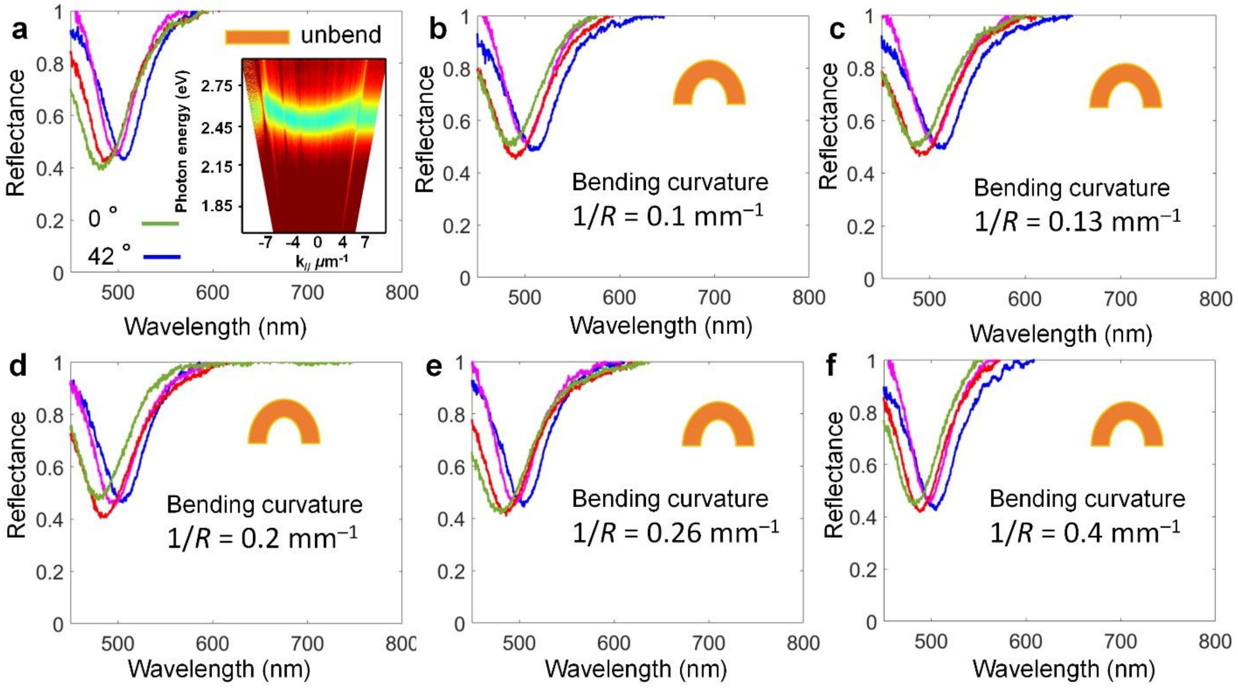

Figure S3. Experiment angle-resolved reflectance measurement of Ag/Al_2O_3/mica multilayers films under bending state with the incident angle changes from the normal plane (0°) to 40°. (a) The experiment angle-resolved reflectance of Ag/Al_2O_3/mica multilayers films without bending.

The inset shows the angle-resolved reflectance mapping of films. (b)-(f) The experiment angle-resolved reflectance of Ag/Al_2O_3/mica multilayers films under bending conditions with 0.1, 0.13, 0.2, 0.26, and 0.4 mm^{-1} bending curvatures.

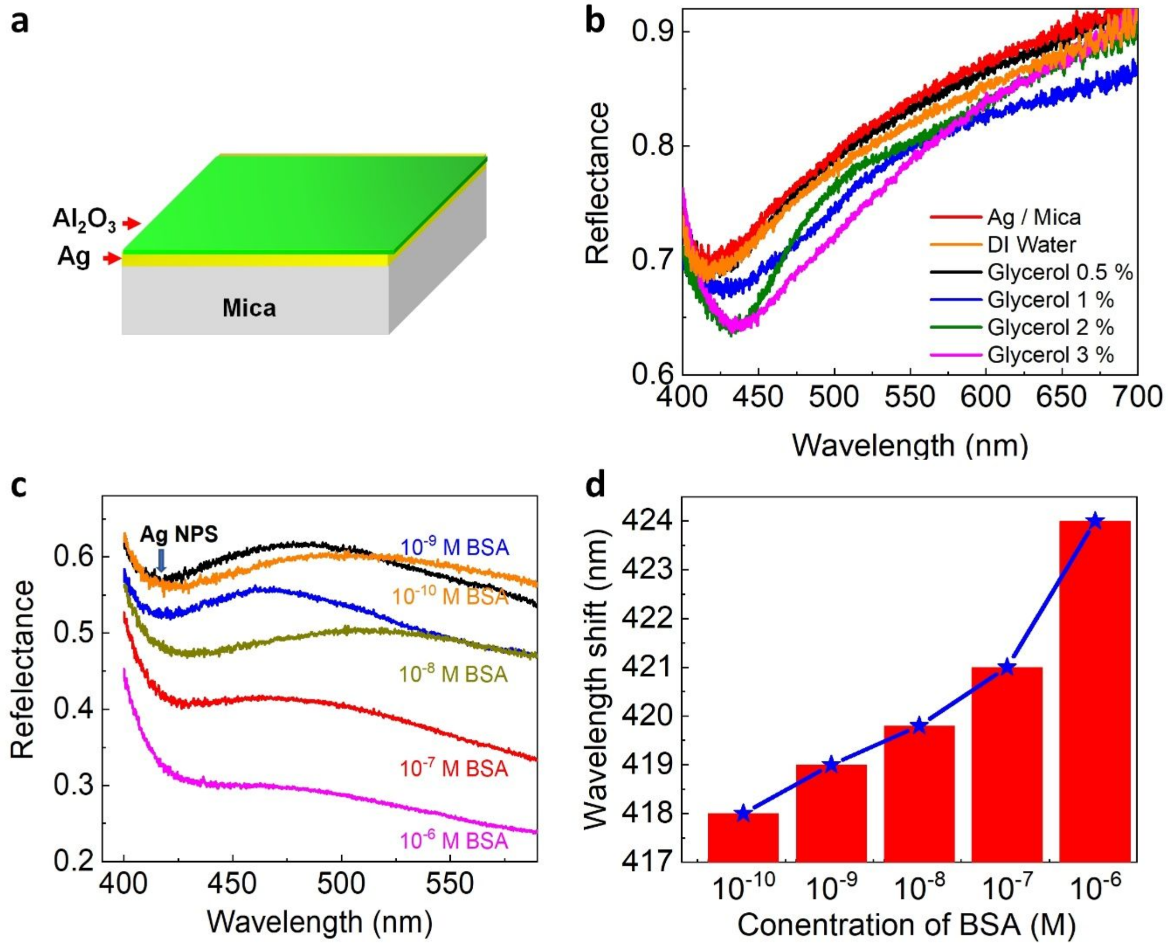

Figure S4. Sensing performance based on pure Ag/mica film. (a) schematic of Ag/mica films. (b) Measured reflectance spectra as a function of glycerol (b) and BSA (c) aqueous solutions. (d) The wavelength shift while the concentration of BSA increased from 10^{-9} to 10^{-6} (M).

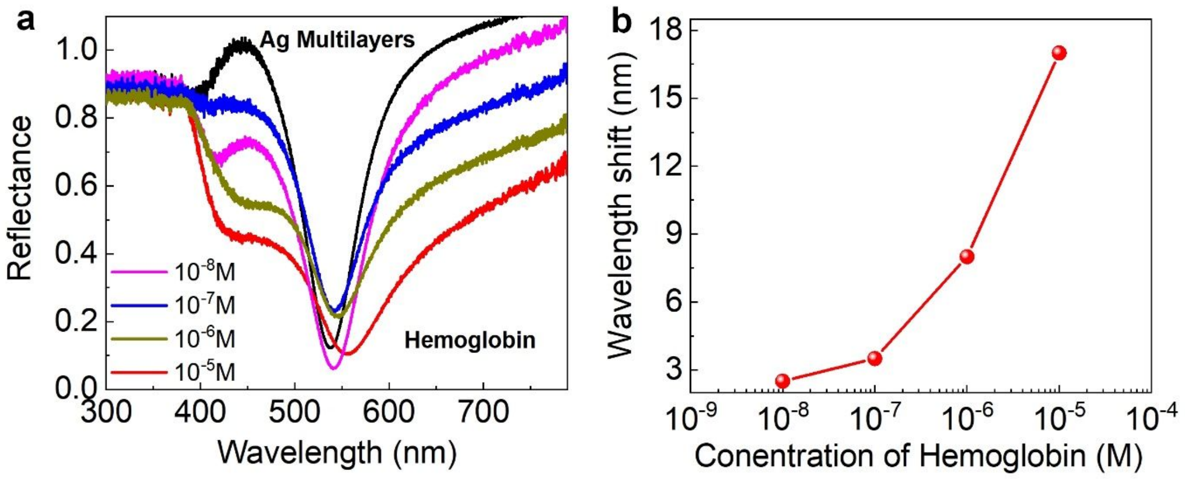

Figure S5. (a) Measured reflectance spectra as a function of hemoglobin solutions. (b) The wavelength shift with different concentration of hemoglobin.

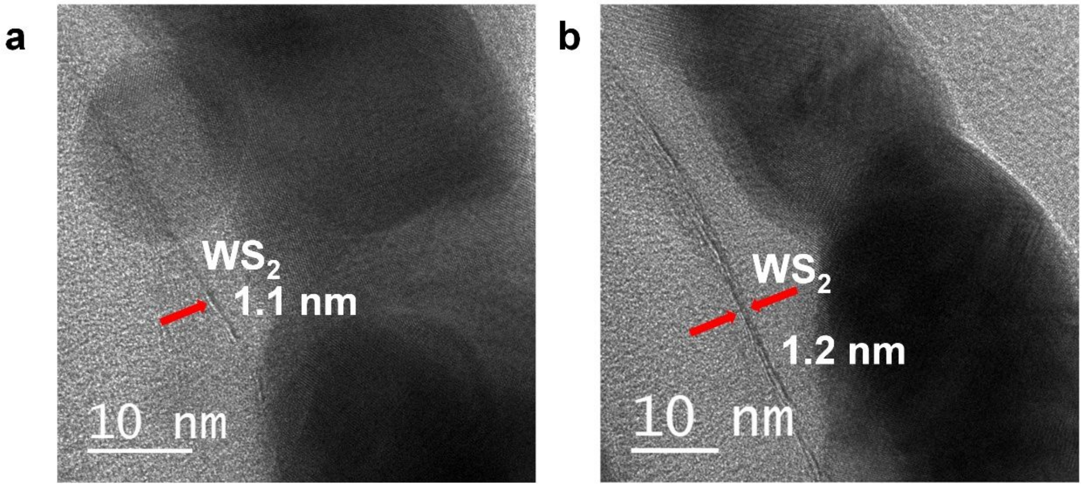

Figure S6. (a) and (b) High-resolution TEM showing a monolayer of WS_2 placed on Ag/Al_2O_3/mica multilayers film.

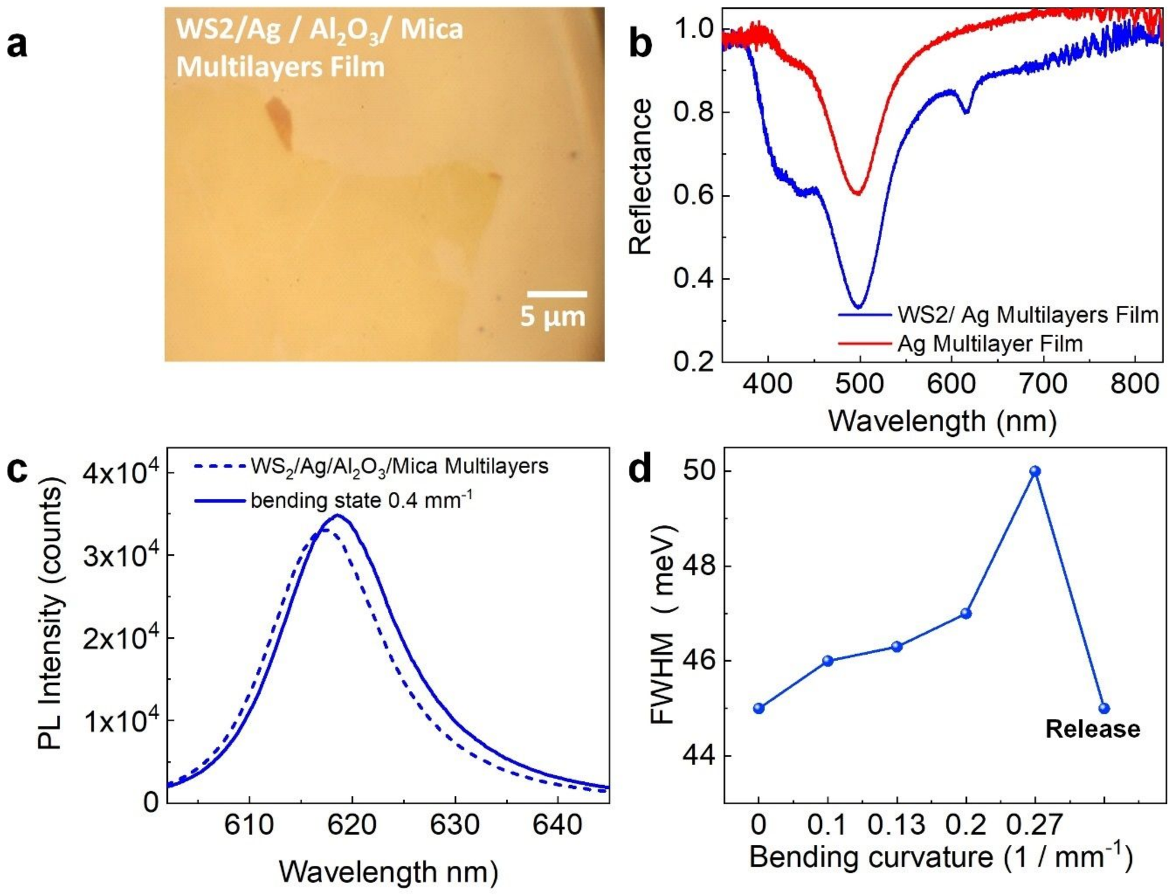

Figure S7. (a) optical image of $WS_2/Ag/Al_2O_3$/mica multilayers film. (b) Reflectance of Ag/Al_2O_3/mica multilayers film and $WS_2/Ag/Al_2O_3$/mica film. (c) PL measurement under

0.4 mm^{-1} bending state and flat sample of $WS_2/Ag/Al_2O_3$/mica multilayers film. (d) full weigh haft maximum (FWHM) of PL peak of WS_2 monolayer under bending state.

The refractive index sensing parameters are defined by

Sensitivity = $|\frac{\Delta\lambda}{\Delta n}|$

Figure of Merit (FOM) = $\frac{\text{sensitivity}}{\text{linewidth}}$ S1

Table S1: The performance of biosensing based Ag/Al_2O_3/mica multilayer films

Wavelength (nm) of SPP at 0 nm	498	**506.6**	**518**	**524.6**	**535**
n_{eff} (0 nm)	1.333				
n_{eff} (0.5 %)		1.3336			
n_{eff} (1 %)			1.3342		
n_{eff} (2 %)				1.3353	
n_{eff} (3 %)					1.3365
**Sensitivity (nm/RIU)**		**14000**	**16833**	**11608**	**10485**
Line width	84	84	84	86	77.4
**FOM**		**167**	**195**	**136**	**135**

Table S2: Comparison of sensing performance based metasurfaces with previously reported works.

**Materials/Structures**	**S (nm/RIU)**	**FOM (RIU^{-1})**	**LOD**	**Wavelength (nm)**	**references**
$Al/SiO_2/Si$	245			1232	S2
Ag: MIM (FDTD)	2320			730	S3
Au/Al_2O_3:8/8 HMMS	30000	590			S4
Au nanograting arrays	547		37pM	480	S5
$Au/TiO_2/Au$	800		10ng/mL	700	S6
Au nanorod arrays	32000		300nM	1280	S7

Ag/Al_2O_3/mica multilayers	16833	194		518	This work

Reference

(S1) Gao, Y.; Gan, Q.; Xin, Z.; Cheng, X.; Bartoli, F. J. Plasmonic Mach-Zehnder Interferometer for Ultrasensitive On-chip Biosensing. *ACS Nano* **2011**, *5*, 9836–9844.

(S2) Ray, D.; Raziman, T. V.; Santschi, C.; Etezadi, D.; Altug, H.; Martin, O. J. F. Hybrid Metal-Dielectric Metasurfaces for Refractive Index Sensing. *Nano Lett.* **2020**, *20* (12), 8752–8759.

(S3) Rakhshani, M. R.; Mansouri-Birjandi, M. A. High Sensitivity Plasmonic Refractive Index Sensing and Its Application for Human Blood Group Identification. *Sensors Actuators, B Chem.* **2017**, *249*, 168–176.

(S4) Sreekanth, K. V.; Alapan, Y.; Elkabbash, M.; Ilker, E.; Hinczewski, M.; Gurkan, U. A.; De Luca, A.; Strangi, G. Extreme Sensitivity Biosensing Platform Based on Hyperbolic Metamaterials. *Nat. Mater.* **2016**, *15* (6), 621–627.

(S5) Arcadio, F; Zeni, L; Montemurro, D; Eramo, C; Ronza, S, D; Perri, C; D'Agostino, G; Chiaretti, G; Porto, G; Cennamo, N. Biochemical Sensing Exploiting Plasmon Sensors Based on Gold Nanogratings and Polymer Optical Fibers. Photonics Research 2021, 9, 1397-1408.

(S6) Hackett, L. P.; Ameen, A.; Li, W.; Dar, F. K.; Goddard, L. L.; Liu, G. L. Spectrometer-Free Plasmonic Biosensing with Metal-Insulator-Metal Nanocup Arrays. *ACS Sensors* **2018**, *3* (2), 290–298.

(S7) Kabashin, A. V.; Evans, P.; Pastkovsky, S.; Hendren, W.; Wurtz, G. A.; Atkinson, R.; Pollard, R.; Podolskiy, V. A.; Zayats, A. V. Plasmonic Nanorod Metamaterials for Biosensing. *Nat. Mater.* **2009**, *8* (11), 867–871.
